# Explaining costly religious practices: credibility enhancing displays and signaling theories

**DOI:** 10.1007/s11229-022-03742-7

**Published:** 2022-06-02

**Authors:** Carl Brusse, Toby Handfield, Kevin J. S. Zollman

**Affiliations:** 1 Department of Philosophy and Charles Perkins Centre, The University of Sydney, Camperdown, NSW 2006 Australia; 2 School of Philosophy, RSSS, The Australian National University, Acton, ACT 2601 Australia; 3 SOPHIS, Monash University, Clayton, VIC 3800 Australia; 4 Department of Philosophy, Carnegie Mellon University, Pittsburgh, PA 15213-3890 USA

**Keywords:** Evolution of religion, Signaling, CREDs, Evolutionary explanation, Cultural evolution

## Abstract

This paper examines and contrasts two closely related evolutionary explanations in human behaviour: signalling theory, and the theory of Credibility Enhancing Displays (CREDs). Both have been proposed to explain costly, dangerous, or otherwise ‘extravagant’ social behaviours, especially in the context of religious belief and practice, and each have spawned significant lines of empirical research. However, the relationship between these two theoretical frameworks is unclear, and research which engages both of them (especially in systematic comparison) is largely absent. In this paper we seek to address this gap at the theoretical level, examining the core differences between the two approaches and prospects and conditions for future empirical testing. We clarify the dynamical and mechanistic bases of signalling and CREDs as explanatory models and contrast the previous uses to which they have been put in the human sciences. Because of idiosyncrasies regarding those uses (especially with signalling), several commonly supposed differences and comparative advantages are actually misleading and not in fact generalisable. We also show that signalling and CREDs theories as explanatory models are not interchangeable (or reducible to one another), because of deep structural differences. As we illustrate, the proposed causal networks of each theory are distinct, with important differences in the endogeneity of various phenomena within each model and their explanatory targets. As a result, they can be seen as complementary rather than in competition. We conclude by surveying the current state of the literature and identifying the differential predictions which could underpin more comprehensive empirical comparison in future research.

## Introduction

Scientific explanations of human behavior are particularly concerned with behaviors which are individually costly and burdensome. Some can be seen as investments toward future personal benefit, such as thatching a roof in order to enjoy a more comfortable winter. Some are costly sacrifices undertaken with the aim of securing a collective good, such as volunteering to fight in intergroup conflict. Yet other behaviors, however, are less clearly explicable in either of these terms. Participation in painful and arduous religious rituals is a prime example. Individuals in various cultures will variously pierce or cut their flesh, walk across hot coals, carry heavy burdens up high peaks, and sacrifice their livestock, as expressions of religious devotion. Although supernatural beliefs may justify these behaviors in terms of anticipated future benefit, from an evolutionary perspective such explanations are suspect. Given that these practices have very real fitness costs, we would expect some countervailing force is required to explain their persistence.

Two frameworks have been particularly influential in recent scientific literature on costly religious behaviors, and costly behaviors more generally: signaling theory (Barker et al., 2019; Bliege Bird et al., 2018; Bulbulia, 2004; Bulbulia & Sosis, 2011; Irons, 2001; Power, 2017; Sosis, 2003, 2005; Sosis & Alcorta, 2003) and the theory of credibility enhancing displays (CREDs) (Henrich, 2009; Lanman & Buhrmester, 2017; Norenzayan et al., 2016; Singh & Henrich, 2020; Willard & Cingl, 2017). While very similar in some respects, the distinction between these two explanatory approaches is illustrative of a deeper divide with respect to evolutionary biology and cultural evolutionary theory; between economic/strategic reasoning on one hand, and psychology and social learning on the other.

Although CREDs were introduced as an alternative to signaling, the relationship between the two approaches is not consistently marked in the literature. The two models are sometimes presented as rivals, or alternatively with CREDs described as merely an extension of ‘mainstream’ signaling theory. This paper contributes to the literature by clarifying the ways in which both approaches are compatible and clearing up some misconceptions about possible limitations of each theory. We then turn to identifying what predictions might differentially falsify/confirm one approach versus the other, with the hope of guiding future efforts at data collection. We focus on religious rituals, in part as a matter of convenience, and in part because religious behavior has been the area in which CREDs theory has been most extensively applied to date.

We argue that part of the confusion here stems from differing assumptions and interpretations with respect to biological versus cultural evolutionary explanations, especially regarding selectionism. The biological-cultural distinction itself contrasts two explanatory domains, while selection vs non-selection concerns modelling strategy. These distinctions do not perfectly line up. In principle, the abstract schema of selection/fitness/heredity could be applied to anything which fits the bill as a unit of selection and obeys selectionist dynamics. At a sufficient level of abstraction, the selectionist schema can be agnostic about the mechanisms which underlie such dynamics, and the question becomes whether it is scientifically accurate, apt, or useful to model a given target system in that way. Cultural evolutionary explanations may or may not be selectionist, depending on the mechanisms involved.

With respect to signals and CREDs, one of our aims in this paper is to tease apart the abstract models (selectionist or not) from their theorist-preferred applications (biological or cultural). In particular, we emphasize that the explicitly selectionist signaling model can be applied to traditional natural selection of biological traits (as in animal signaling), but also to the selection of traits which are differentially propagated by cultural learning mechanisms.

### Application: offertory

The broad distinctions between the two approaches can be illustrated with an example of a widespread and familiar religious practice: offertory, in traditional Christian church services the collection of alms, or similar ritual donations (and similar practices in many other religions), where churchgoers make offerings of money into collection boxes, plates, or bags during or after the service. Such offerings come at an obvious cost to those who make them (oblationers), but they are also very much public acts: churchgoers observe each other making offerings and (to some extent) how much.

One hypothetical signaling interpretation of this case (there are several to choose from) might go as follows. Offertory serves as a costly signal of cooperative disposition or commitment by the oblationer (taking the role of the sender) to their community, i.e. the other churchgoers (the receivers of the signal, when observing the donations their compatriots). Having observed each other’s signals, receivers tend to treat generous senders more favorably in the future than they would otherwise. This might come in the form of beneficial cooperation, social opportunities, upgraded status and trust, or in the form of avoiding the withdrawal of such social goods (or even punishment) for failing to perform the signal. Importantly, these benefits may be more valuable to some than to others: those who are not sufficiently committed to living among the community according to their ways will not find the benefits worth the cost of the signal. This differential benefit ensures that sufficiently costly signals tend to be reliable. Communication is thereby achieved: the practice of offertory can serve as a reliable, incentivized signal of commitment that weeds out flakes and freeloaders, facilitating the positive assortment of community-minded individuals.

In general, signaling interpretations seek to explain rituals as solutions to problems of cooperation/coordination, whereby likeminded communities can come together for some mutual benefit (in this interpretation, altruistic cooperation).

The most basic formal representations of signaling theories are static strategic games, which do not make predictions about how people adopt new strategies (i.e. ritual behaviors), but merely predict what combinations of strategies are stable.

A CREDs interpretation, in contrast, is both less explicitly strategic and more explicitly dynamical. It is an account of how cultural learners choose to adopt new behaviors and beliefs from those currently extant in the population. It does not focus on the mutually beneficial exchange of information or strategic interaction, but rather represents ritual practices as competing in a marketplace of behaviors to be imitated and reproduced via social learning mechanisms. Given all the other things one could do, why do people imitate the behavior of offertory in particular? CREDs assumes that people learn by imitating certain others (models) who appear knowledgeable and successful. But to the extent that oblationers are seen to become marginally poorer than those who do not give their money to the church, such cases are still prima facie puzzling. People do not merely choose to imitate behaviors, however, they also learn what to *believe* by social learning. The CREDs model allows that complementary packages of belief and behavior can prove particularly attractive to learn. First, belief that God eventually rewards pious behavior makes the burdens of pious behavior easier to tolerate. Second, engaging in pious behavior serves as a potent demonstration of such a genuine belief in the promise of heavenly rewards. In combination then, an offering’s cost serves as a way of making both the offering and its motivating beliefs more credible to observers. Observers will be led to think that oblationers must really believe what they profess to believe (why else would they part with their money so willingly?), lowering the barrier of credulity for those beliefs and making them more attractive to imitate. And having adopted those beliefs, they will find it attractive to imitate the practice of offertory because the beliefs justify the practice. The belief and practice reinforce one another, and the costs associated with the practice, seen as willingly carried out, make them both more salient and more compelling in the context of social learning.^1^

In short, signaling theory says that costly behaviors persist because they communicate useful information. CREDs theory explains the persistence of costly behaviors as parts of particularly attractive packages to imitate.

### Signaling and CREDs as models

Both modeling approaches use equilibrium modelling, in that they formalize tendencies toward religious practice/belief over time under idealized conditions (payoffs, biases, etc.). And both can be seen as what Levins called ‘type 3’ population models, which sacrifice precision for sake of realism and generality (Levins, 1966). This means that while they predict that *some* beliefs and practices should be pervasive (under the right conditions), neither are equipped to predict the origins or outcomes of any specific real-world examples. Likewise, neither are falsified by a lack of rigid, equilibrium-like stability among these phenomena, which are largely afloat on a sea of exogenous economic, demographic, and cultural change.

Both approaches therefore idealize away much of the detail of human cultural/religious life and focus on limited sets of putatively important variables and parameters (including traits and environmental features). These sets partially overlap, which we detail more fully in Sect. 3. However, some of the focal differences are relatively straightforward.

For example, because CRED models focus on the imitation of beliefs as well as practices, they essentially involve representational content and cognitive traits. This makes these models especially apt for organisms with rich mentalizing capacities, like humans, and for explaining tight correlations between complex beliefs and behaviors. Signaling models, in contrast, are applicable to humans and non-humans alike, and need not make mention of cognitive states like beliefs. For instance, it strains credulity to say a peahen has any *beliefs* about the fitness of a peacock based on observation of his tail, but this is no obstacle to a signaling explanation for the tail. Likewise, with an appropriate interpretation of costs and benefits, a signaling model might be able to explain offertory even if none of the churchgoers were genuine believers, as long as the practice still served its function of filtering for socially desirable community members. The content of signals can be entirely distinct from their function, and communicative signaling may be variously underpinned by ostensive sincerity, hypocrisy, or unthinking reflex.

Despite the distinct causal mechanisms being posited, the relationship between these two explanatory approaches is not always clear. For example, in Henrich’s initial, highly cited 2009 paper, he describes an evolutionary pathway for CREDs using orthodox signaling terminology (Henrich, 2009, p. 255), but frames the view as a more promising alternative to “the ritual signaling hypothesis”, which might nevertheless co-exist with it. Some theorists have explicitly described CRED theory as just another version or ‘extension’ of signaling theory (Bulbulia & Sosis, 2011; Wildman & Sosis, 2011), while others argue for a sharp distinction (Brusse, 2020). Recent empirical studies of religious practice have tended to engage with either signaling or CRED explanations to the exclusion of each other, rather than in comparison or combination (Barker et al., 2019; Lanman & Buhrmester, 2017; Power, 2017; Willard et al., 2016).^2^ One outlier in this regard is a recent field study of how adherence to religious restrictions influences the perceptions of religious leaders in a traditional population in Indonesia, testing the putative predictions of CREDs and commitment signaling (Singh & Henrich, 2020). As the authors point out, asceticism and other religious self-denying behaviors might serve as signals and CREDs simultaneously, and they see both sets of predictions borne out by their results.^3^

Our first goal in this paper is to elaborate on the formal and conceptual differences between the two models, and to demonstrate how the models, though distinct, are not in direct competition with one another, in fact being complementary for many purposes. Notwithstanding that, we then consider a number of suggested ways in which one approach might be superior to the other—at least for the purposes of explaining costly religious behaviors—and argue that none of these have much substance. There are no knockdown philosophical reasons to favor one over the other, and both approaches are a reasonable fit with existing data. Finally, to move beyond this impasse, we identify crucial differences in the predictions that may be supported by each model. Future research addressed to these predictions is most likely to differentially falsify or confirm one approach or the other.

## The models and their uses

### Signaling models

Signaling models in biology and economics usually take the form of game-theoretic ‘signaling games’ played between senders and receivers. Senders can perform an action (make a move) based on some state of the world otherwise hidden from the receiver. Receivers can make their move based on their observation of the sender’s move. The receiver’s move, in the context of the actual state of the world, determines “payoffs” for both sender and receiver (with the sender perhaps paying some cost for their signal). “Payoff” here can refer to fitness, utility, or any other quantity that plays the functional role of predicting the choices of sender and receiver. The payoff structure determines which strategy combinations constitute stable equilibria of the game.

To this static picture, *evolutionary* game theory adds dynamical processes whereby players update their strategies to improve their expected payoffs. This dynamical element explains which of several equilibria are more likely to evolve, are more stable, and so-forth. Such dynamics might correspond to mechanisms of biological evolution, cultural evolution (through processes like imitation), or individual learning; signaling models per se are largely agnostic with respect to the details of the dynamical mechanism. Within this framework there is an array of modelling permutations with potential application to a wide variety of contexts.

We will limit ourselves to cases where the hidden information concerns a trait of the sender. The trait can be a relatively fixed quality such as physical strength, wealth, or ancestral lineage, but it may also be a dispositional type that is relevant for predicting future behavior—such as a tendency to selfishness or generosity. If the sender’s signal is conditionalized on the sender’s trait (different trait types send different signals), and the receiver’s move is conditionalized on the signal (they behave differently when they receive different signals) then actionable information is being communicated by the sender and acted on by the receiver. If the incentives are appropriately aligned, this sort of scenario can constitute a “separating” equilibrium, in which neither sender nor receivers would do better by switching to an alternative, unconditional strategy (Skyrms, 2010). The offertory case we considered was an example of this: the favor of a community is extended to those who make costly offerings, because only those who are worthy of the community’s favor find it valuable enough to be worth the cost. This can be contrasted with “pooling” equilibria, where the incentives are instead such that (i) senders do not correlate their signal with the observed trait (e.g. *everyone* signals that they trustworthy), or (ii) receivers do not correlate response with signal (e.g. no one is trusted, regardless of what they do).

In some signaling games sender and receiver have clearly aligned interests, and signals allow them to solve a coordination problem. For instance, a motorist and a pedestrian communicating with each other to work out who will give way: both are interested in avoiding a collision. Although communication is not guaranteed in such cases, it is relatively easy to achieve (Bruner et al., 2014). As an illustration, an example of religious practices that rely on common interest might look like this: People have an interest in interacting with others who have similar expectations about marriage, child-rearing, and domestic divisions of labor (Sterelny, 2007, Sect. 5). Religious practices serve as a quick and easy mechanism to communicate a complex bundle of cultural norms that relate to these domains, and so serve a coordination function.

Not all signaling games presume common interest, however. In other signaling games, the parties have partially conflicting interests. A job applicant wants to be hired, regardless of their quality; but the employer only wants to hire the applicant if they are sufficiently diligent—how can a truly diligent applicant convincingly signal to the employer that they have this trait? Broadly speaking, when the interests of sender and receiver are not strictly aligned, some mechanism must be present to prevent deceptive signals from undermining communication. The classic mechanism is a differential in signal costs which disincentivizes dishonest signaling. A job applicant who has completed a difficult degree course while juggling a full-time job has done something that is difficult for all applicants, but is much easier if the applicant is in fact diligent. This may serve then as a signal of quality to the employer, and because of its cost it is much more credible than merely asserting “I will work hard”. Cases such as this were used in foundational work on signaling in economics (Spence, 1973), with similar reasoning employed to articulate ‘handicap’ signaling theory in biology (Zahavi, 1975).

Similar ideas are widely used to explain a variety of human behaviors which might otherwise appear gratuitously costly, both in contemporary society and in the archaeological record (see e.g. Quinn, 2019). These include culturally localized behaviors such as dueling (Allen & Reed, 2006), restrictions of female freedom (Rai & Sengupta, 2013), honor killings (Thrasher & Handfield, 2018), terrorism and political violence (Hoffman & McCormick, 2004; Lapan & Sandler, 1993; Pape, 2006), as well as broad categories of behavior such as aggression (Frank, 1988), grief (Winegard et al., 2014) and regret (Rosenstock & O’Connor, 2018). Others have argued that relatively costly activities widely assumed to be worthwhile, but for which the direct evidence of their success is often underwhelming (such as higher education and healthcare), can be explained as instances of costly signaling (Caplan, 2018; Hanson, 2008). Even behaviors that appear to be deliberately avoiding flamboyance, such as small, hard to observe fashion logos or anonymous donations, may be amenable to a signaling explanation (Hoffman et al., 2018). As one of the most ubiquitous, traditional, and puzzling features of human societies however, religion has been treated as something of a special target (Brusse, 2020).

In Sect. 1.1 we described one way of constructing a religious signaling hypothesis, using one particular model (differential benefit costly signaling) applied to one aspect of religious practice (offertory) which putatively signals for a desirable trait (community commitment). This is one among many possible combinations of models, target systems/signals, valued traits, and so-forth; we will not seek to summarize them here.^4^

One point to clarify though is that the essential logic of signaling games does not always require or predict the presence of costly signals. Signaling only requires differential payoffs for high-type and low-type senders (i.e. those who possess the desired trait, and those who do not) such that only honest signaling is incentivized (Hurd, 1995; Lachmann et al., 2001; Számadó, 2011). For example, high type signals could have negligible cost as long as excessive signal costs for low types outweighed any possible rewards; in such a signaling game, the only signaling which occurs in equilibrium should therefore be costless (i.e. by high types only). An example may be obtaining a tattoo or other indelible mark of group identity, which should have no adverse consequences if one remains a loyal member of the group, but which will cause one to be killed if they try to emigrate to an out-group (Skarbek, 2014 discusses similar phenomenon in the context of US prison gang tattoos). Because of those potential costs, the tattoo may be a reliable indicator of one’s tendency to remain: only high types (the solidly loyal with no desire to migrate) will obtain the tattoo.^5^

The costly/strategic signaling framework is flexible enough to also include some fairly trivial cases where signal costs are non-differential or entirely non-existent, even out of equilibrium. Again, what is required for a signaling equilibrium is that the net payoff for signaling is positive for high-types and negative for low-types. So differential *benefits* can do the job just as well as differential signal costs (Johnstone, 1997). For example, two potential applicants for initiation into a religious society could be presented with the same up-front costs, but have different cost–benefit calculations based on their commitment levels and how attractive membership is to them (Iannaccone, 1992). In this case, participating in flat-cost initiation rituals can serve as a reliable signal of actually wanting to be a long-term member of a community (as opposed to just exploiting it until moving on) (Brusse, 2020; Iannaccone, 1994).^6^

It should also be remembered that signaling models are compatible with failures of communication: if the cost–benefit schedule of the signal does not effectively discriminate between high and low types, a population may arrive at a pooling equilibrium where receivers only cooperate in the presence of the signal, making it a pre-requisite for interaction, but both high and low types send the signal, rendering it meaningless. This sort of equilibrium is less stable than a separating equilibrium, because the receivers would obtain an equivalent payoff if they stopped treating the signal as meaningful. But in the absence of any perturbation to receiver strategies, the meaningless signal will continue to be incentivized. Such pooling equilibria are less interesting in the context of explaining communication, but evidently can still contribute to explaining the persistence of a gratuitously costly behavior.

While most of our examples in this section have focused on signaling a discrete property that takes on two values (one is either a high- or low-type), signaling models can account for more complex signaling. The types can take on as many values as one likes, the signal can be graded, and the response can be very complex. For our purposes, none of these extensions are necessary, but one should not suppose that they are excluded by signaling models.

Signaling models thus have the potential to explain a wide variety of display-type behaviors: costly as well as costless, discrete as well as continuous, and meaningful as well as meaningless cases.

### Credibility enhancing displays

The CREDs approach is less dependent on the communicative value of the displays it seeks to explain, and also far less heterogeneous in its range of possible applications. First introduced in (Henrich, 2009), it models a joint distribution of belief and practice over a population, where the belief is one which “justifies” the practice. To take one of Henrich’s examples, consider (i) the belief that blue mushrooms are safe to eat, and (ii) the practice of actually eating blue mushrooms. Obviously, someone who has the belief has better reason to engage in the practice, and someone who engages in the practice can more credibly assert the belief. Other examples might include belief in a deity and worshipping that deity; belief that an area is good for hunting and actually hunting there; belief that a behavior is taboo and abstaining from that behavior.

The purpose of the model is to predict or explain the distribution of belief and practice that will evolve in a population that has a given learning process. The particular puzzle to be explained is why social learning sometimes leads to widely practiced costly behaviors. The fundamental learning process is the imitation of successful types, so the beliefs and practices of the populous and successful will proliferate. More precisely, the probability that an agent will imitate a given trait is proportional, both to the frequency of that trait and also to the relative “success” of those who have the trait. In addition to these familiar foundations (Richerson & Boyd, 2005), Henrich’s CRED model introduces additional elements in the learning process, based on two intuitions: (i) that “actions speak louder than words”, and (ii) that people are more likely to adopt a costly practice if they believe that the practice has benefits—even if that belief is mistaken. Putting these elements together, the learning dynamic is characterized by the following points:

- *Frequency dependence* Beliefs are more likely to be learned the more other people have the belief. The same goes for practices. This is because of the sheer weight of numbers: other things being equal, a learner will be more likely to observe a common practice rather than a rare one, and hence be more likely to imitate the common one.^7^
- *Success following* Practices and beliefs are both more likely to be learned if they are seen to be success-enhancing. Learners are more impressed by people who seem to be “successful”—healthy, not sick; prosperous, not poor; etc.—and are more inclined to imitate them.
- *Credibility enhancement* Beliefs are more likely to be adopted if the model is non-hypocritical: that is, the model has practices consistent with the professed belief. Someone who claims that blue mushrooms are safe to eat but refuses to eat them herself is less credible and less likely to be imitated.
- *Interaction between belief and valuation* A learner will perceive the practice to be less costly if they have a belief that “justifies” the practice. So the likelihood that an individual will imitate a practice depends not just on how common the practice is, nor on how objectively successful the practitioner is, but on whether the learner believes the practice to be costly. Believing that the mushroom will not kill me makes me more likely to eat the mushroom, even if it is in fact poisonous; and vice versa.

Provided the cost of the practice is not too great, and granting some modest restrictions on the model’s free parameters, then the CRED model gives rise to two equilibria: one in which everyone rejects both the practice and the belief, and another in which everyone adopts both the practice and the belief. There is a tipping point, such that once a sufficient proportion of the population has the belief and sufficiently many adopt the practice, the population will evolve to the belief-plus-practice equilibrium, thereby stabilizing a gratuitously costly practice in the culture.

We should emphasize that these equilibrium states may be quite dissimilar from those of a classical signaling model, at least in principle. This is largely because the mechanism of stabilization, while sensitive to excessive burden, does not require that costly display practices end up being compensated for by the benefits of a cooperative equilibrium. Indeed, CREDs can potentially help explain cultural forms that are strictly maladaptive at the individual level.

## Comparing the models

### Explanatory targets

We can now more precisely isolate the differences between the two modelling approaches, by returning to the offertory example described earlier. To contrast the two approaches under conditions of maximum overlap, we have chosen an alternative signaling interpretation in which the *underlying trait* is belief in God, which is also the *belief* to be imitated (or not) in the CRED model. With respect to this signaling interpretation, we assume that the receiver finds subjective value in not being fooled, while all senders, regardless of type, would prefer to be treated as if they are true believers. This might be because true believers make better cooperative partners in joint projects, or for any of a wide range of reasons. We further assume that the cost of the offering constitutes the credibility-enhancing feature of the display, for CRED purposes.^8^

**Fig. 1:**
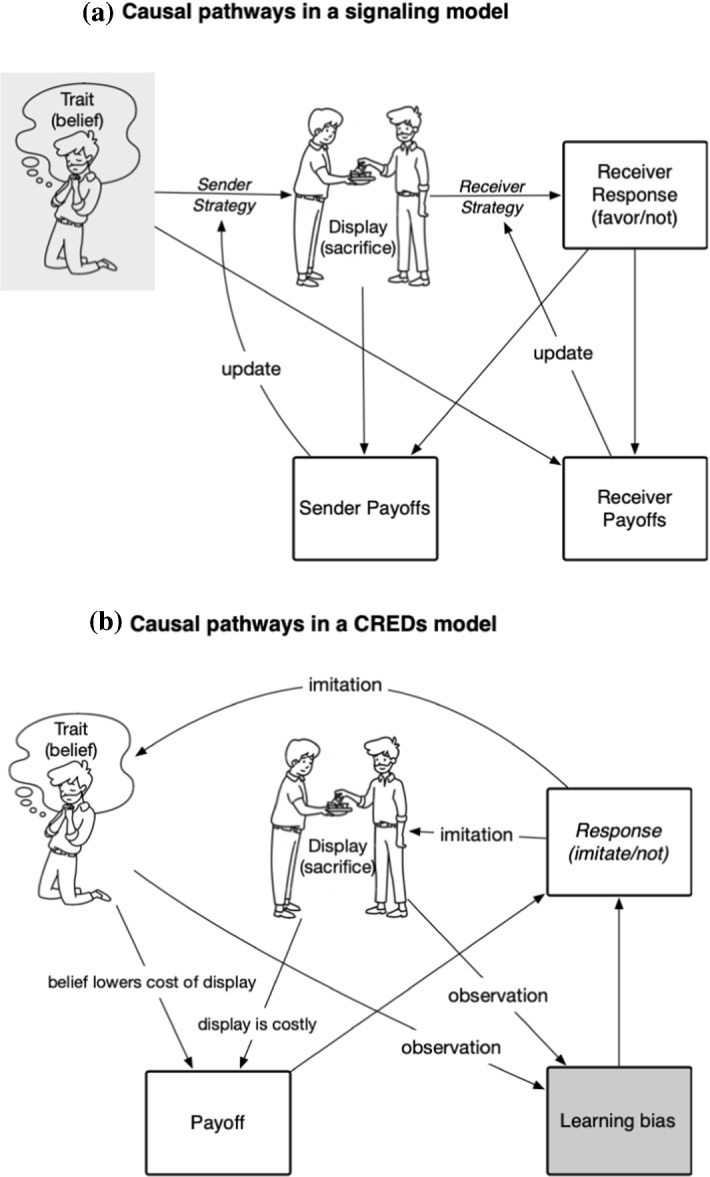
Schematic illustration of the causal pathways involved in signaling models and in CRED accounts. In each case, the principal variables to be explained are italicized: in the signaling model, it is the sender and receiver strategies. In the CRED model, it is the “response”—imitation or not of the display and the trait. (We omit for clarity an additional causal arrow which goes from the frequency of the trait and the display to response.) Key differences we wish to highlight are: (i) the location of exogenous factors (indicated by shaded nodes): in signaling models the distribution of the trait is exogenous; in CREDs, the magnitude of the learning bias is exogenous; and (ii) that there is no strategic interaction modelled in CREDs—everyone receives the same payoffs and those payoffs are not changed by the choices of others; whereas in signaling the strategy of receiver and sender are distinct, interdependent, and coevolve. Also evident is the central similarity of the two models: both explain the distribution of the costly display.

Figure 1 illustrates the commonalities and differences between the two explanations, by way of the parallel, partially overlapping causal processes that they posit. In this figure, the features common to both models are the underlying sender trait (being a believer, or not) and the costly display (making an offering or not).

In Fig. 1a is the looping feedback process posited by signaling. Both parts of the communication process—the choice whether to display, and the choice whether to respond—are influenced by the payoffs that come from the receiver’s responses. Payoffs reflect the value agents of each type derive from the outcome of their interaction, and the way the agents update their strategies is assumed to be sensitive to this. We leave unspecified the mechanism by which strategies are updated, because signaling models are compatible with a wide variety of mechanisms.

In Fig. 1b we see that a CREDs account involves very similar variables. Here, the distribution of the “trait”—belief in God—is endogenous to the model, i.e. an evolving variable which it describes. The learning bias which makes agents prefer to imitate the beliefs of non-hypocritical agents is a fixed parameter or assumption (exogenous). Also importantly different is that in the CRED model, the population is modelled by a single payoff function—there is no strategic asymmetry between senders and receivers. The key mechanism of the model is in the links between combinations of belief and display and subsequent decisions to imitate both of those: believers are more likely to perform the display (because the belief justifies it); credibility enhancements make receivers more likely to imitate those who both profess belief and adopt the practice, and payoffs affect imitation decisions because those who are more successful are more likely to be imitated.

We should reiterate that the interpretation of costs and benefits we use here is not the only signaling-based explanation available. Likewise, CRED models might well explain imitation of beliefs which would not be relevant traits for signaling purposes. The key functional role of the belief, for the purposes of CREDs, is that the belief is seen to justify the costly practice. The key functional role of the trait, for the purposes of signaling, is that receivers prefer different sorts of interactions with senders, depending on whether or not they possess the trait. Obviously these two functional roles can come apart.

Even having contrived our example to maximize the overlap between the models, there are important differences in the proposed mechanism of action. The key differences are (a) the trait/belief being endogenous to the CREDs model but exogenous to signaling, and (b) the phenomena being directly modified by the respective causal mechanisms of each model: the belief, display, and response for CREDs, and the behavioral strategies in the case of signaling.

So, the CRED approach models the evolution of belief (trait) and display (signal) as endogenous variables, while the learning biases which operate on them are exogenous features of human psychology. Signaling instead explains what strategies agents use to both send and receive information—which in effect endogenizes the learning process that is exogenous in CREDs. The frequency of the underlying trait—being a believer or not—is, however, exogenous to the signaling model: the receiver’s response is a *filter* for sender types and does not itself alter the distribution of types in the population. This of course leaves changes in trait frequency unexplained by signaling evolution alone (cf. Henrich, 2009, pp. 255–256). In other words, this signaling model assumes some people are true believers, and explains how others can identify them via costly displays, despite the possibility of deception. CREDS, on the other hand, assumes that costly displays are regarded as more trustworthy—and uses this assumption to explain the frequency of true believers in the population.

Additionally, the causal mechanisms posited by the two explanations differ. Favoring and following are different—treating the sender as a welcome cooperation partner is different from imitating them. This is where predictions of the models most clearly diverge.

Because of these differences in explanatory target, there is no contradiction between the two causal models: even when (as in our interpretation), we contrive to bring them as closely into contact as possible. They operate in parallel on different features of the overall phenomena, and could easily be complementary, non-antagonistic processes within a single causal system. This would be especially true for signaling interpretations which appealed to cultural selection rather than just natural selection, with CREDs taking responsibility for direction of change for the belief trait, and both working in concert on stabilizing or proliferating the offertory display.

It is reassuring that while the models are somewhat similar, they are nonetheless distinct. In the next section, we turn to addressing some of the alleged reasons to prefer one theory or the other.

### Is one theory better for explaining costly religious behavior?

Notwithstanding their compatibility, there has been some suggestion that CRED models have an advantage over signaling models in explaining phenomena such as religious belief and ritual, based on supposed differences between the approaches (see especially Henrich, 2009, p. 256). We have already considered some of these in passing, however, many supposed differences rest on mistaken or overly narrow characterizations. For example, Henrich argued that signaling explanations must predict that signaling displays should be significantly more costly for nonbelievers to perform than for believers, which is not especially plausible in the religious context. The only difference between believers and non-believers is a psychological distinction with negligible direct fitness implications when it comes to sacrificing resources, or risking health or security. As outlined previously though, signaling models are compatible with observed signaling costs being equivalent for high and low types by appeal to a variety of mechanisms: (i) coordination signaling—where there is no conflict of interest between parties, (ii) high counterfactual costs absent in equilibrium but which disincentivize signals for low types, and (iii) differential benefits rather than differential costs.

Other putative differences that have been mooted as reasons to prefer CRED models include:

- CRED models focus on psychological causation and cultural evolution, rather than natural selection and fitness, and therefore might be better placed to explain:
  - phenomena like the distribution of belief traits (Norenzayan, 2013),
  - the evolution and ‘ratchetting up’ of religious traits over rapid, intra-generational timescales (e.g. Henrich, 2009).
- CRED models might have an advantage over signaling models in explaining how costly religious practices can solve collective action problems (Henrich, 2009)

In the rest of this section we review these lines of reasoning, and argue that some are incorrect and none are compelling reasons to think the CRED framework is generally superior.

#### The importance of modelling belief

In some presentations of religious signaling, it has been suggested that there may be a genetic component to the evolution of sender and receiver behaviors (e.g. Bulbulia, 2013). It has been argued that the CREDs approach would have an advantage in this domain, because costly religious practices are not universal, and the diversity of beliefs and practices that needs to be explained is better suited to a cultural evolutionary account (Norenzayan, 2013, p. 102).

This argument assumes too much; conflating selectionist explanation with biological domain, as outlined at the introduction of this paper. Advocates of religious signaling often borrow the biological language of selection, fitness, and adaptive behavior, but *natural* selection is not central to the signaling approach itself. Certainly, in Spence’s Nobel-prize winning work on costly signaling in economics, the strategies of senders and receivers are volitional or learned behaviors, with the ‘fitness’ and ‘selection’ roles in the models being performed by economic utility and rational choice, respectively (Spence, 1973). Signaling explanations posit or assume: (i) payoff structures for sender/receiver behaviors to define the signaling games, and (ii) the updating of player strategies in the direction of improved payoff. For much non-human animal communication, the payoff and update roles are played by biological fitness and natural selection. But in human cases the payoff may be. purely subjective benefit (psychological utility) and the update mechanism some sort of social learning such as “follow success” bias (Boyd & Henrich, 2002; Boyd & Richerson, 1985). Indeed, when comparing simulation experiments using update dynamics designed to mirror biological natural selection on one hand and social or individual learning on the other, the results are so similar that the simpler biological models can safely be used for most applications (Beggs, 2005; Börgers et al., 2004; Schlag, 1998).

So, while advocates of CREDs are correct that cultural transmission is better suited to explaining belief, this is not in conflict with signaling theory. What signaling requires is that the transmission mechanism be sensitive to payoff success (as in the success-following principle used in various cultural evolution frameworks). In principle, signaling theory can therefore help itself to some of the same cultural learning mechanisms that CREDs appeals to. The fact that signaling theory is typically expressed in the terminology and formalisms of animal signaling implies no necessary commitment to biologically ‘hard-wired’ signaling strategies.

Three things remain true though. First, (as illustrated in Fig. 1) the content of religious beliefs in themselves is not a primary explanatory target in signaling explanations. In CRED accounts, because beliefs need to psychologically justify the costly practice, the content of those beliefs is essential to the explanation. In a signaling model, beliefs are only functionally important to the extent that they influence payoff-relevant behaviors or strategy change (cf. Hall et al., 2015, p. 1375). Whether this instrumental attitude toward belief is intellectually satisfying or not is arguably an aesthetic choice rather than a modelling one, though perhaps we might also expect a sharp divergence here along disciplinary lines (e.g. from within ‘psychological’ or ‘sociological’ traditions in the science of religion).

Second, while CRED accounts model the change in belief and behavior traits endogenously, a signaling model by itself only explains their positive assortment: it is a model for social filtering and assortment, not trait change directly. That said, it is not ad hoc to expect change to follow, because altering the assortment of desirable social traits will of course alter their expected payoffs/fitnesses. For this reason, some approaches combine the evolution of signaling strategies with the independent evolution of the frequency of high types (e.g. Gintis et al., 2001).

Finally, it also remains true that signaling, at least in its classical, ‘adaptive’ formulation used in the science of religion by Bulbulia and Sosis, posits a cost–benefit analysis which explains signaling as being adaptive at the *individual level*, while this is not a necessary feature of a CREDs model. Credibility-enhancing displays and the practices they justify can be strictly maladaptive at the individual level and still proliferate, depending on other model parameters and additional details, such as the possibility of cultural group selection. As discussed though, empirical predictions and comparisons should respect the fact that a signaling model’s payoffs need not be restricted to the domain of reproductive fitness.^9^

#### Solving collective action problems

In Sect. 1, we presented signaling and CRED models as alternative ways of explaining cultural behaviors that appear gratuitously costly, because they do not appear to have corresponding individual or collective benefits. As should now be clear, signaling models explain these behaviors by suggesting that this appearance is in some sense misleading: the costliness of the behavior is not gratuitous, because it delivers some strategic benefit. The equilibrium in which senders and receivers find themselves may or may not be collectively optimal, but it is one in which no one has an incentive to unilaterally change their behavior. In some cases, these equilibria are collectively beneficial: cooperative solutions to a social dilemma.

Notwithstanding that signaling has an inherent connection to explaining cooperation, it has been suggested that for some of the most puzzling forms of human cooperation, CRED models have an explanatory advantage over signaling. Henrich argues that if the credibility enhancing action is one that is pro-social in its effects, or if the action is one that increases the sense of group identity and solidarity, it can bootstrap a solution to the public goods problem.^10^

Henrich further claims that, in contrast, there is no model to show how signaling can solve a prisoner’s dilemma with more than two agents (Henrich, 2009, p. 256). Both the accuracy and the relevance of this claim is questionable.

Evolutionary models of cost-free signaling *do* show ‘secret handshakes’ can arise which enable pairwise cooperation (Robson, 1990; Santos et al., 2011). While Henrich’s critique focuses on multi-player social dilemmas, it is not clear why he thinks these kinds of results could not be extended to these settings. In fact, some early work has been done on how signaling contributes to evolution of cooperation in multi-player public goods problems (Pacheco et al., 2015).

There are also models showing that indirect reciprocity can sustain cooperation in a number of collective action problems, (See Okada, 2020 for a recent review) and in the n-person prisoner’s dilemma in particular (Suzuki & Akiyama, 2007). Indirect reciprocity is a mechanism whereby cooperative behaviors earn the cooperator a favorable public reputation, which will be rewarded by inspiring cooperation from others in future (Nowak & Sigmund, 2005). Although explicitly modeling this as a signaling game would be problematic, there is a strong functional resemblance: when cooperation evolves via indirect reciprocity, agents in effect use costly patterns of cooperation as signals of underlying behavioral type, which only pay off if the responses are favorable. Related models and experiments have suggested that willingness to police cooperative norms by engaging in punishment of defectors may also be a costly signal of cooperative traits (Jordan et al., 2016). In a very different vein, Zahavi and Zahavi suggested that contribution to public goods might itself function as a signal of another trait, like mate quality (Zahavi & Zahavi, 1997). Similar ideas relating to the value of displaying altruism as a signal of quality in a biological market have been developed under the label “competitive altruism” (Barclay, 2004; van Vugt et al., 2007).

These signaling games are, in some respects, different from the canonical signaling game we focus on here, and more work needs to be done to show that they are plausible explanations of human cooperation. They all share, however, the idea that different types of agents may be distinguished on the basis of costly behaviors they undertake, thus allowing more cooperative types to assort together, overcoming a cooperative dilemma. This is the essence of how signaling promises to solve collective action problems.

It is also worth noting that one-shot public goods problems may not be the right problems to solve, in order to explain the high level of cooperation observed in humans. Because most interactions are repeated—especially in the ancestral environment, and because of extensive opportunities for mutualism, our key cooperative encounters for understanding the evolution of cooperation are arguably better understood as either assurance problems or coordination problems (Sterelny, 2012, Chap. 1). These problems are not without their challenges, but they are much less difficult scenarios in which to achieve cooperation, because the conflict between individual and collective interest is less acute. Signaling can have an even greater effect on cooperation in these games (Pacheco et al., 2015; Santos et al., 2011; Skyrms, 2004).

### Future research: differential predictions

Grose (2011), following Searcy and Nowicky (2005), argues that signaling models make three key predictions regarding a separating equilibrium.

1. Receivers respond to signals.
2. The signal is reliable. In other words, the signal covaries with the trait of interest.
3. The reliability of the signal is explained by the correlation between the marginal cost/benefit of the signal and the trait of interest.

From first to last, these predictions are increasingly difficult to empirically confirm. Evidence consistent with the first has been documented on several occasions, and includes findings that people have more favorable attitudes towards those who wear religious badges (McCullough et al., 2016), make costly donations (Hall et al., 2015), attend synagogue frequently (Ruffle & Sosis, 2007; Sosis & Ruffle, 2003), or observe dietary and sexual taboos (Singh & Henrich, 2020).

The second prediction requires that those who give the signal actually possess a trait which makes them preferable interaction partners compared to those who do not signal. Of the studies cited above, only one provides such evidence (Ruffle & Sosis, 2007): those who attend synagogue more frequently are more cooperative in an incentive compatible economic game.

The third prediction is the hardest to demonstrate, for at least two reasons. First, the relevant costs and benefits are often difficult to operationalize. If we assume (somewhat implausibly) that a signaling equilibrium has emerged via natural selection, the relevant costs and benefits should be impacts on biological fitness, which are rarely feasible to measure (though see Shaver et al., 2020 who address fertility benefits of ritual participation, which may be relevant). In the biological literature, usually a suitable proxy is found such as energetic expenditure or predation probabilities (e.g. Møller et al., 1998). These proxies are both empirically and ethically more fraught in the case of humans.

If we instead assume that some sort of cultural selection is involved, we can instead assume that signaling has different *psychological* costs/benefits at the margin for different types. The challenge then is to avoid trivializing the prediction: if we assume everyone is a rational utility maximizer, it follows that for signalers, the benefits of signaling must outweigh the costs, and the opposite must hold for non-signalers. But that is exactly the prediction we are trying to confirm, so this assumption is stultifying to any attempt at empirical falsification.

The functional role of utility in cultural selection models is not exhausted, however, by the assumption that individuals maximize their expected utility. It is also essential that *third parties* be able to estimate the psychological cost/benefit of various behaviors in order to choose whether to imitate a given belief or practice. So data on third party perceptions of the costliness of behaviors is at least a good beginning—but it is essential to ask for perceptions of how costs may vary by type. We are aware of no data of this sort to date.

Additionally, because this prediction relates to the cost/benefit of marginal signaling behavior, it is important to make observations out of equilibrium: how much would a high type lose by failing to signal, and how much would a low type gain if they were to signal? Since in equilibrium high types do signal and low types do not, to identify these marginal effects requires an exogenous shock to force low types to signal, or to deprive high types of their signal. In animal studies, for instance, long tail feathers have been attached to males with short tails, to assess the additional mating opportunities they are afforded and decreased aerodynamic performance suffered due to this forced alteration to their signaling presentation (Andersson, 1982; Rowe et al., 2001). Making such interventions without introducing new confounds, however, is very difficult. We are aware of no human research that has addressed this difficulty. Singh and Henrich (2020) come closer than most when they present data on the perceived cost of various potential signals, but they rely on third party observations of typical cost, rather than obtaining estimates that speak to whether the costs differ between types.

Notwithstanding the difficulty in confirming the third prediction of a signaling model, with the second prediction we already have the potential to differentially confirm a signaling theory as opposed to CREDs. A signaling theory predicts that—at least in some circumstances—a costly behavior will systematically covary with a cooperative trait. CREDs, by contrast, never makes this prediction, simply because cooperative traits are not within the ambit of the CRED theory (see Table 1 below).

**Table 1:** The distinctive patterns of signal, trait, and cost that are explained by a signaling model, as opposed to a CRED model, (where there is a partial conflict of interest).

	Signal does not correlate with trait	Signal correlates with trait
Cost/benefit of signaling does not differentiate types	CREDs and signals	Neither
Cost/benefit of signaling differentiates types	CREDs	Signals

Only a signaling theory explains why a signal may correlate with a trait of strategic interest, but it does so only in the case where the cost/benefit structure of the signal is appropriate. Note that neither theory is strictly inconsistent with the quadrants from which it is absent, merely that they cannot explain observations in those quadrants as stable equilibria

Put bluntly, the CRED mechanism does not, on its own, explain any observed correlation between cooperative traits and costly behaviors. This might seem surprising, given CREDs have been suggested as integral to the explanation of how moralizing big god concepts might have been instrumental in promoting high levels of human cooperation (Norenzayan, 2013; Norenzayan et al., 2016). As noted above, these explanations succeed because they make an additional posit: that the belief which justifies the costly practice promotes cooperative behavior. The special nature of the belief in a “big god” is serving to underwrite the key correlation required of a signaling model: that there is a correlation between a costly practice and cooperative behavior. In this way, CRED models can come to overlap quite closely some of the predictions of a signaling model, but in virtue of an exogenous assumption. To this extent then, any evidence that religious signals promote cooperation is not in conflict with a CREDs model, but more directly confirms a signaling approach.

In contrast, to differentially confirm a CRED theory, we could look for cases where costly behaviors become widespread in a community without any apparent impact on strategic interactions. This is obviously difficult, given that it requires establishing an absence of interdependence of costs and benefits, and human life is rife with strategic implications. But perhaps future research may focus on relatively personal areas of behavior, such as hygiene, diet, medicine, and sleep practices. Another area for distinctive corroboration of CREDs is in the learning biases that it posits. The prior probability for most of these posits, however, should be relatively high. It is common sense psychology that actions speak louder than words, and that we are less likely to believe hypocrites who say one thing but do another; but any evidence disconfirming these ideas would be a non-trivial threat to the CRED account.

A more specific prediction derived from CREDs has been empirically confirmed in a recent paper (Kraft-Todd et al., 2018): the tendency to imitate the practices of others is mediated by second-order beliefs — beliefs about what the model believes. As mentioned above, signaling models make no such predictions because they do not include any specific claims about psychological representations.

To reinforce our conclusion that there is a dearth of evidence that can be used to adjudicate between the signaling and CRED approaches, in Table 2 we present a sample of papers that investigate behaviors that might be explained by signaling or CRED accounts and comment on their suitability for confirming either type of theory. Although the majority of papers approach their topic in terms of signaling, it is very rare that any papers gather data on strategically relevant traits that might be subject to communication, or on marginal costs/benefits of signaling. Further, on the occasions when data relevant to signaling is obtained, it is very rare that any attempt is made to check predictions relevant to the CRED framework. This analysis is not intended as a criticism of the existing work but in the hope that it will make vivid to future researchers what sorts of evidence are required to advance our comparative understanding of the two models.

**Table 2:** A comparison of papers on costly religious displays, comparing the evidential relevance of these papers for both signaling and CRED models

Paper and theoretical framework	Display Behavior	Costs/benefits of display behavior	Strategically relevant trait of “sender”	Beliefs as potential targets of imitation	Comment
Hall et al. (2015) Framed as example of signaling	Donations to religious charities, observing dietary restrictions	Not measured	Third-person beliefs about trustworthiness	Religious beliefs observed, but only at one time, so no opportunity to observe change in beliefs	Study finds costly religious behaviors are perceived as signals of trustworthiness, across a religious divide. No evidence obtained to test the reliability of the signals. No evidence obtained to test the propensity to imitate religious beliefs
Iannaccone (1994) Describes multiple causal processes, relevant to both signaling and CREDs	Compliance with various burdensome religious requirements (some but not all of which are inherently altruistic)	Descriptive comparison based on ethnographic/sociological data, surveys of expert opinion	Level of religious commitment, exemplified e.g. by church attendance	General Social Survey data on belief in afterlife and belief in religious literalism correlated with strictness of religious practice	Basic logic of the paper is to test signaling, but evidence on beliefs is also congenial to CRED model
Lanman and Buhrmester (2017) CRED-specific investigation	Religious participation/sacrifice	Costs implicitly measured by survey of third party perceptions of participation/sacrifice. (Offspring estimating parental sacrifice.)	No strategic interaction studied	Dependent variable is theism/religious belief of offspring	Shows that parent’s CRED-like behavior is more relevant to predicting religious belief in children than profession of religious belief. More directly relevant to issue of cultural transmission of religious belief
McCullough et al. (2016) Signaling	Religious badges, specifically Christian symbols (arguably these are not costly displays, except to extent they may have led to stigmatization)	Measure third party willingness to invest in trust game with signalers, which is some indicator of marginal benefits to signaler. But signalers were fictitious, so cannot identify covariation of benefit and trait	Measure third person beliefs about traits	Measured religious beliefs of receivers, but as a covariate for dependent variable. Not concerned with changes in belief	Study comes closer than many to showing real strategic payoff for signaling, but the effect on trust game behavior is not statistically significant
Power (2017) Primarily signaling (though makes reference to CREDs also)	Variety of religious behaviors, not just dramatic rituals	Tries to take into account the costliness of different acts, but no attempt to test whether the cost/benefit differs with differing traits	Third person beliefs about variety of prosocial traits	Third person beliefs about “devoutness” of others measured, but does not try to explain imitation of belief	Finds costly behaviors are effective signals, and identifies distinct trait/signal correlations
Purzycki and Arakchaa (2013) Signaling	Religious observance	No measure of costs	Measure third person beliefs about traits	Not measured	Observations consistent with costly religious observance as a signal of trustworthiness, within an ethno-religious group
Shaver (2018) Signaling—though not with emphasis on costly signals	Religious badges in Mauritius, crossed with ethnic appearance	Third party willingness to invest in economic trust game; third party estimates of general trustworthiness	Not measured	Not investigated	Similar study to McCullough et al. ad Hall et al., but finds less clear evidence that religious badges are signals of trust across religious groups. Also suggests interaction effect with ethnic markers
Shaver et al. (2020) Signaling—with emphasis on fertility benefits of religious signaling	Religious observance (frequency of attendance at church or similar)	Several variables of interest studied: Social network support; Aid from coreligionists; Fertility; Child development outcomes	Not measured	Not investigated	Documents potential fitness benefits from religious displays. But restricts attention to benefit received; no investigation of cooperation/aid given by signaler
Singh and Henrich (2020) CREDs and signals	Observance of food taboos, and sexual abstinence	Reports variation in benefits obtained by signalers, but no correlation of benefit level with levels of signaling On cost: measure third person estimates of average cost, thus no individual difference data available to correlate with signaling	Measure third person beliefs about traits	Not measured. Though see related work on transmission of religious ideology (Singh et al., 2020)	Find no evidence that type of self-denial affects inferred trait. (Cf. Power, 2017.)
Soler (2012) Signaling	Variety of religious behaviors	Questionnaire also asks about cooperation received by signaler. Finds marginally significant result that more signals sent predicts more benefits received Also measures “vulnerability”. Those who need cooperation more, are predicted to signal more. Mixed findings in this regard	Public goods game play; self report of helping behavior given	Not measured	Treats CREDs together with all manner of signaling theories, but author implies that cost is essential. No citations to papers on idea of no-cost signals Makes an effort to remove index signals—so all religious behaviors that are inherently cooperative were removed. N.B. These are the sort of behavior that Henrich suggests enable CREDs to solve public goods problems
Sosis and Ruffle (2003), Ruffle and Sosis (2007) Signaling	Synagogue attendance, communal dining	Model assumes constant cost, variable benefit. No attempt made to empirically vindicate assumption regarding differential benefits, except insofar as that preference is revealed by differential behavior in the economic game	Cooperation in common resource game (public goods game with a taking frame)	Not measured at individual level, though comparisons are made between religious and secular kibbutzim. No mechanism of belief transmission studied	Find contextually appropriate costly signals (synagogue attendance in a religious kibbutz, communal dining in a secular kibbutz) are correlated with altruistic behavior in a public goods game. Has some data on migration into the community which may be relevant for explaining distribution of cooperative traits within the kibbutz
Willard and Cingl (2017) CRED focused. Use a CRED scale developed by Lanman and Buhrmester	CRED scale: Attending church, volunteering, behaving fairly, being “pure”, avoiding harm to others, making sacrifices	Not measured	Not measured	Religious participation in the next generation	Compares Czech Republic and Slovakia, two countries with shared history, but divergent trajectories of religious observance. Finds that observation of costly religious behaviors in earlier generations highly predictive of religious belief in later generations. No evidence relevant to signaling analyzed
Xygalatas et al. (2013) Signaling and CREDs, no differentiation by authors. Though see comments for ways in which the evidence perhaps favors CREDs	Performing Kavadi: a high-intensity, painful religious ritual	Painfulness measured using subjective report. Those who perceived Kavadi as more painful (either as observers or as performers) donated more. Authors do not note this, but this finding is prima facie at odds with signaling theory: if the signal is supposed to be less costly for those with the trait, those who donate should experience less pain. Note, however, that as per the discussion above, the key prediction occurs out of equilibrium: for those who are *not* performing Kavadi, if they did perform they should find it even more painful (or should expect less benefits from subsequent cooperation)	Anonymous donations of money earned in experiment	Potentially interesting data on social identity gathered for primary participants, but no belief transmission studied	Norenzayan (2013) says this is a good example of CREDS, because those who merely observe the painful ritual are more likely to donate more to the temple. This is only an indirect test, however, because no belief transmission was measured

## Conclusion

We have argued that for many purposes, CRED accounts and signaling models do not directly compete. Although both can explain costly behaviors (religious and otherwise), they have different emphases which are not strictly incompatible. CREDs assumes that people prefer to learn what to believe from people who demonstrate their sincerity; this in turn explains the high prevalence of beliefs which justify costly practices; and that in turn explains the high prevalence of costly practices. Signaling models take the distribution of beliefs (or more generally traits) for granted, but explain why we find costly practices to often be a reliable indicator of what one believes. Signaling thus explains costly practices as ways of solving a communicative problem. Obviously, both these accounts could be true of the same phenomenon.

Although both theories have been fruitfully applied to understanding aspects of religious behavior, we have also argued that little or no attention has been paid to obtaining data which would simultaneously be germane to the predictions of both theories. We hope that by clarifying the issues at stake in each theory, we have provided guidance for future researchers who might hope to undertake more targeted interrogation of the two accounts.

## Footnotes

1 It should be noted that the cognitive bias for seeing sincerity as more imitation-worthy (all else being equal) is a basic assumption of the CRED model. This will be plausible in contexts where the belief is readily checked and/or has pragmatic consequences; we don’t want to be tricked into eating poisonous foods, planting sickly crops in barren lands, etc. It may be that this bias extends much less to the context where the beliefs have no readily verifiable content, or fitness impacts. However, it is also true that nothing will select against a bias in these cases.

2 Though we will not explore this in detail, it is possible that there is a disciplinary divide at play here, with anthropologists and social researchers dealing in the ecology of behavior gravitating to signaling explanations, and the more proximate, individual-level psychological mechanisms of CRED theory appealing to a different subset of researchers. This is suggested by the different conceptions of religion held by the founding fathers of psychology and sociology. William James defined religion as: “The feelings, acts, and experiences of individual men in their solitude, so far as they apprehend themselves to stand in relation to whatever they may consider the divine” (James, 1902). Durkheim however saw it as “a unified system of beliefs and practices relative to sacred things, that is to say things set apart and forbidden—beliefs and practices which unite into one single moral community called a church, all those who adhere to them.” (Durkheim, 1915). It is easy to imagine that foundational assumptions about the subject matter can influence the approaches taken to explaining it.

3 Even in this study, however, the empirical predictions are open to debate. For example, the authors predict that signaling should lead to self-denying religious leaders being explicitly *perceived* as more cooperative by the lay population, but this prediction assumes that signaling is cognitively transparent—that receivers understand the underlying prosocial information communicated by the signal. As mentioned, a signal can function without either sender or receiver being cognizant of the underlying information being signaled. All that is necessary is an increased likelihood of fruitful positive assortment due to the signal, and this could be achieved by any reliable response which produces the right result.

4 For a broader taxonomy see (Brusse, 2019, 2020).

5 Arguably, even the high types must pay some modest expected cost here, in terms of a non-zero risk that they will find it hard to join a new group if their group collapses due to some exogenous shock. If such events are suitably common/predictable, it might indeed be appropriate to factor such risk into the payoffs of a signaling game. The point remains though that if we ignore exogenous/counterfactual disruption, there are no observable signal costs *in equilibrium*, contra the predictions of a traditional “handicap” account of costly signaling. For relevant discussions of the costs of indelible signals see (Skarbek, 2014; Sterelny, 2020).

6 A further way that honest signals might be costless is via *index* signals, where signal honesty is guaranteed by physical or biological constraints on the signal-generating mechanism, rather than disincentives for lying. The canonical example of this is the roar of a stag, which provides a reliable, hard-to-fake index of the stag’s size and vigor: small stags are physically incapable of making a loud roar (Maynard-Smith and Harper 2003). We omit further discussion of this case from the paper, because it is of limited application in the comparison of CRED and signaling models.

7 Note that this density effect is distinct from learners being strong conformists (which involves perceiving the prevalence of traits in the overall population), though greater conformity bias will achieve the same result faster.

8 In the context of religious CREDs, the belief in a moralizing God should have a relatively robust impact on one’s trustworthiness, which is a highly relevant trait for followers (Norenzayan, 2013). Thus, in these settings there is a particularly salient bridge between belief and trait. We are not explicitly endorsing the signaling interpretation here, but it follows a well-known model of religious costly signals proposed by (Sosis, 2003).

9 We do however restrict ourselves here to signaling interpretations where selection based on payoff is carried out at the level of the individual: as a biological organism, economic or rational agent, etc. It would of course be possible to embed a signaling model within a cultural evolutionary framework such that the cultural fitnesses of the beliefs/behaviours themselves are being modelled. This may greatly blur the lines between these modelling approaches but again is not part of the consideration here.

10 Some recent experimental evidence has been offered in support of this hypothesis (Kraft-Todd et al., 2018).
